# Effects of bariatric surgery on drug pharmacokinetics—Preclinical studies

**DOI:** 10.3389/fphar.2023.1133415

**Published:** 2023-04-05

**Authors:** Angela Mercado, Anna Pham, Zhijun Wang, Wendong Huang, Patrick Chan, Hajer Ibrahim, Hyma Gogineni, Ying Huang, Jeffrey Wang

**Affiliations:** ^1^ College of Pharmacy, Western University of Health Sciences, Pomona, CA, United States; ^2^ College of Pharmacy, Marshall B. Ketchum University, Fullerton, CA, United States; ^3^ Department of Diabetes Complications and Metabolism, Arthur Riggs-Diabetes and Metabolism Research Institute, City of Hope National Medical Center, Duarte, CA, United States; ^4^ Kaiser Permanente, San Jose, CA, United States

**Keywords:** bariatric surgery, gastric bypass, pharmacokinetics, obesity, rodent model

## Abstract

With the rising worldwide obesity rates, bariatric surgeries are increasing. Although the surgery offers an effective treatment option for weight loss, the procedure causes dramatic physiological and metabolic changes. Animal models in rodents provide a valuable tool for studying the systemic effects of the surgery. Since the surgery may significantly influence the pharmacokinetic properties of medications, animal studies should provide essential insight into mechanisms underlying changes in how the body handles the drug. This review summarizes research work in rodents regarding the impact of standard bariatric procedures on pharmacokinetics. A qualitative literature search was conducted *via* PubMed, the Cochrane Central Register of Controlled Trials (CENTRAL), and EMBASE. Studies that examined bariatric surgery’s effects on drug pharmacokinetics in rodent models were included. Clinical studies and studies not involving drug interventions were excluded. A total of 15 studies were identified and assessed in this review. These studies demonstrate the possible impact of bariatric surgery on drug absorption, distribution, metabolism, excretion, and potential mechanisms. Pharmacokinetic changes exhibited in the limited pre-clinical studies highlight a need for further investigation to fully understand the impact and mechanism of bariatric surgery on drug responses.

## Introduction

According to the World Health Organization, worldwide obesity rates have nearly tripled between 1975 and 2016. As of 2016, more than 1.96 billion adults aged 18 years and older were overweight, with 650 million of these adults falling into the category of obese (asmbs, 2019). Obesity has been associated with many health conditions and leading causes of death, including heart disease, stroke, diabetes, and some cancers (CDC, 2022). When obese patients fail to respond to dietary and lifestyle modulations coupled with pharmacological interventions, bariatric surgery can be considered. A large body of evidence has demonstrated that bariatric surgery for the treatment of obesity reduces all-cause mortality as well as obesity-associated morbidity (Wiggins et al., 2020).

Bariatric surgical procedures result in weight loss *via* two primary mechanisms: restriction and malabsorption. Consequently, it has been found that the surgery drastically decreases the gastrointestinal (GI) surface area, thus, affecting the absorption of nutrients and drugs in the longer portion of the GI tract. As most nutrient absorption occurs in the small intestine, bariatric surgery can affect drug absorption due to the loss of mucosal exposure. Bypassing a portion of the small intestine can also alter drug metabolism as it is one of the body’s sites of first-pass metabolism due to its large amount of CYP3A4 enzymes.

Oral administration of medications has been the preferred route of drug delivery due to high compliance and adherence of patients as well as lower costs. This fact alone demonstrates a significant need for investigation into the effect of bariatric surgeries on the pharmacokinetics of medications. Pre-clinical rodent models have been shown to replicate human bariatric surgery and can be a great asset in investigating pharmacokinetics and pharmacodynamics of pharmacological interventions (Tichansky et al., 2008; Bueter et al., 2012). This review summarizes current pre-clinical findings and the implications of physiological and metabolic changes post-bariatric surgery on oral drug bioavailability.

## Methods

We searched the following electronic resources for studies examining bariatric surgery’s effects on pharmacokinetics and pharmacodynamics in animal studies: PubMed, The Cochrane Central Register of Controlled Trials (CENTRAL), and EMBASE. Search terms used included (but were not limited to): bariatric surgery, gastric bypass, RYGB, Roux-en-Y gastric bypass, rats, mice, drug absorption, gastrointestinal absorption, medication absorption, bioavailability, metabolism, excretion, distribution, and pharmacokinetics. The search is considered up to date as of November 2021.

We included studies that examined bariatric surgery’s effects on pharmacokinetics and those that had implications that could affect the oral bioavailability of pharmacological interventions. Exclusion criteria included non-animal-based studies, review articles, and those that did not focus on pharmacokinetic changes.

Two reviewers reviewed all search results, performed study selection, and excluded irrelevant articles by independently examining study abstracts for inclusion. Reviewers also cross-referenced article references to identify additional studies for inclusion.

A total of 15 studies were identified that met our criteria. These studies focused on physiological or metabolic changes post-surgery or exhibited identifiable pharmacokinetic changes.

## Results and discussion

Bariatric surgery leads to direct alteration in the physiology of the gastrointestinal system affecting not only nutrient absorption but can also lead to alterations in the absorption of orally administered pharmacological therapies (Sawaya et al., 2012). However, due to the lack of studies on the pharmacokinetics of such therapies, there are no clear guidelines for dose adjustments post-surgery. Current pre-clinical models have illustrated the effects of bariatric surgeries on physiological and metabolic processes that may affect oral drug absorption and metabolism.

### Usage and types of animal models for bariatric surgery

Bariatric surgery has been used for weight control for several decades and has become increasingly popular in humans. The physiological mechanisms of the effect on weight loss have also gained more attention in recent years. Obesity can be categorized as obese with cardiometabolic risk factors and obese without cardiometabolic risk factors. Similar to that of lean individuals, obese individuals without cardiometabolic risk factors have reduced risks of morbidity and mortality (Ahima and Lazar, 2013). In clinical studies, bariatric surgery is not only associated with weight loss but improved cardiometabolic risk factors and mortality (Carlsson et al., 2020). Two most common types of surgeries performed in patients include Roux-en-Y gastric bypass (RYGB) and Vertical sleeve gastrectomy (VSG) often precedes significant weight loss and can persist despite weight regain, suggesting metabolic benefits stem from more complex mechanisms than simple weight reduction. Some patients experience insufficient weight loss or weight regain after bariatric surgery leading to revisional surgery, reflecting the gap in understanding the factors that promote the best metabolic response, as well as the identification of adjunctive therapies that improve the durability of the response to bariatric surgeries (Batterham and Cummings, 2016; Aliakbarian et al., 2020; Heinberg et al., 2020; Andalib et al., 2021). The underlying mechanisms and complex integrated physiological systems to metabolic changes remain incompletely understood. In obese individuals with cardiometabolic risk factors owing to increased peripheral and hepatic insulin resistance, the key driver of insulin resistance is adipose tissue inflammation, specifically in the visceral adipose tissue contributing to the development of type 2 diabetes and metabolic syndrome. In addition, the gut microbiome has long been described as altered in obese with cardiometabolic risk factors (Longo et al., 2019). This suggests that the connection between the microbiome and the immune system is central to regulating visceral adipose tissue inflammation and metabolic health. Obese patients who undergo bariatric surgery are more likely to be on multiple medications for cardiometabolic risk factors and other health conditions.

However, the physiological mechanisms underlying post-bariatric surgery changes still need to be elucidated. Therefore, animal models are still valuable tools that can help investigate these mechanisms and offer advantages over human clinical studies. Animal studies provide more objective and less biased data. For example, while collecting food intake data from humans heavily relies on verbal reports and dietary recall measures, animals can provide more accurate and quantitative data. Additionally, the availability of genetic knockout models is another advantage. Studies have shown that understanding the connection between gut hormones, microbiome, and obesity could lead to new and more specific therapeutic interventions for severe obesity and related health issues, both surgical and non-surgical. By targeting specific genes against specific gut hormones or their receptors, researchers can differentiate between associative and causative relationships of proposed mechanisms of bariatric surgery (Finelli et al., 2014). Animal models can provide a controlled experimental setting to study specific physiological processes and mechanisms that may be difficult or impossible to study in humans. In addition, animal models can help identify potential complications and allow a better understanding of bariatric surgery to improve or develop new surgical techniques (Lutz and Bueter, 2016).

**FIGURE 1:**
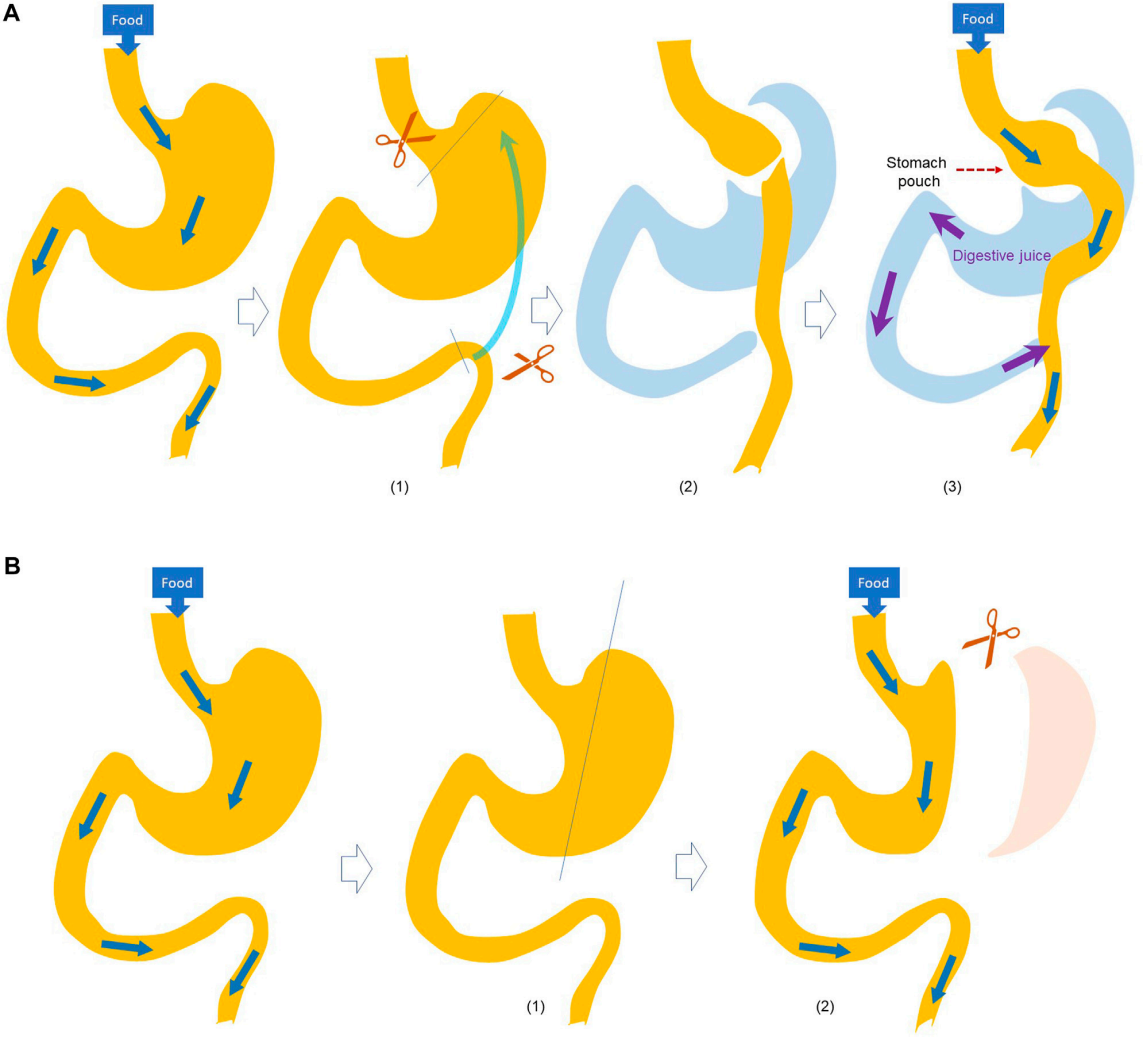
Schematic illustration of **(A)** Roux-en-Y Gastric Bypass (RYGB) and **(B)** SGx surgery. RYGB (asmbs, 2019): A small stomach pouch is made (CDC, 2022); The small intestine is divided and brought up (Wiggins et al., 2020); the small intestine is connected to the small stomach pouch, and the stomach end of divided intestine is connected to the small intestine to allow digestive juice mixing with the food. SGx (asmbs, 2019): A catheter is passed thought the mouth into the duodenum, and (CDC, 2022) a stomach is divided to crate a narrow gastric sleeve.

Many studies have used animal models to investigate the systemic effects of bariatric surgery over the past few decades, with those in rodents, i.e., mice and rats, being the most prominent. Of these rodent models, the most used have been RYGB and sleeve gastrectomy (Ashrafian et al., 2010; Rao et al., 2010) (Figure 1).

RYGB is a restrictive and malabsorptive bariatric procedure. In RYGB, a portion of the stomach is used to create a small gastric pouch, allowing the digestive system to bypass the larger portion of the stomach and a portion of the small intestine (Rêgo et al., 2010). These portions are no longer used in the storage or processing of food, leading to decreased caloric consumption and absorption (Rao et al., 2010).

Sleeve gastrectomy is another form of restrictive procedure where a portion of the stomach is removed to limit the amount of food intake.

Of these models for bariatric surgery, the RYGB model was the most prominent in the studies we identified, which is not surprising as it is also one of the most used in humans.

### Effects of bariatric surgeries on pharmacokinetics

Pharmacokinetics is the study of drug absorption, distribution, metabolism, and excretion. The pharmacokinetic and pharmacodynamic properties of the drugs are highly dependent on physiological process such as gastric emptying, gut mycobiome and systemic metabolism. Physiological and pathological changes caused by bariatric surgery may alter the pharmacokinetics of many drugs. As the prevalence of bariatric surgery continues to rise, the issue becomes more problematic as more individuals will face the issue of potentially requiring medication dose adjustment to reach better therapeutic effects. To assess these alterations, animal studies offer the ability to perform repeated blood sampling and tissue samples which are limited in human studies.

### The impact of bariatric surgery on absorption

Oral drug absorption is a complex process affected by the physicochemical properties of the drug itself and the physiological properties of the person taking it. A myriad of physiological processes is involved in drug delivery, including gastric emptying, pH, small intestinal transit time, bile salts, and metabolism by gut microbiota (Song et al., 2023). Bariatric surgeries in pre-clinical studies have been shown to lead to alterations in bacterial and systemic metabolism (Seyfried et al., 2014; Kaufman et al., 2019), gut microbiota (Shao et al., 2017; Tu et al., 2022), sodium and water handling (Bueter et al., 2012), as well as gastrointestinal hormone concentrations (Suzuki et al., 2005; Kohli et al., 2013; Canales et al., 2014; Prada-Oliveira et al., 2019).

Alteration of the anatomy of the intestine can affect the absorption of nutrients. The proximal small intestine is the primary site for the absorption of nutrients, while there was lower absorption of nutrients in the distal portion of the ileum (Tessier et al., 2019). After RYGB surgery, the proximal small intestine would be removed from the nutrient flow, and thus, it likely contributed to the malabsorption of nutrients and some medications. A study in RYGB rats showed that the removal of the proximal small intestine could cause the impairment of transcellular calcium absorption. Ingested calcium is primarily absorbed in the small intestine (RYGB, 2019), and the sodium bicarbonate exchanger (NHE3) is involved in passive calcium absorption. Removing the proximal small intestine could lead to reduced calcium absorption (Abegg et al., 2013).

Other studies have also shown that bariatric surgery resulted in calcium and vitamin D malabsorption, which has led to bone loss and high serum hyperparathyroidism (Rêgo et al., 2010; Tessier et al., 2019). Data suggested the possibility of the suppression of bone formation, which indicates that future preventions of bone loss are a potential target for patients undergoing bariatric surgery.

Gastrointestinal hormones such as peptide YY (PYY) and glucagon-like-peptide-1 (GLP-1) play a significant role in gastric emptying, motility, gastric acid secretion, and water and electrolyte absorption in the colon. All of which can increase or decrease the absorption of therapeutic drugs. Multiple studies have shown elevated levels of PYY and subsequent increases in gastric emptying times (Suzuki et al., 2005; Canales et al., 2014). GLP-1 levels differ in response to different models of gastric bypass. Suzuki and co-workers showed elevation in PYY post-RYGB, while no changes were observed in GLP-1 (Suzuki et al., 2005). However, other researchers observed increased GLP-1 levels after IR50 bypass and BPD, respectively (Kohli et al., 2013; Prada-Oliveira et al., 2019).

Bile salts are steroid acids that can increase drug solubility and permeability. Changes in bacterial and systemic metabolism of bile salts can affect oral drug absorption. RYGB has been shown to cause larger shifts in the gut microbiome compared to sleeve gastrectomy (Shao et al., 2017). Alterations in gut microbiota have been seen in post-bariatric surgeries, while the magnitude seems to depend on the type of bypass received. At the same time, it has also shown decreases in gut microbiota diversity. These alterations may implicate changes in oral drug absorption as gut microbiota are responsible for the metabolism of some drugs and have been shown to affect pharmacologic efficacy and safety through alterations in drug bioavailability or activity.

Silymarin is a mixture of flavonolignans, with silibinin as the most abundant and active ingredient. It has been used to treat hepatic steatosis in non-alcoholic fatty liver disease patients. Its pharmacokinetics have been conducted in RYGB Sprague-Dawley rats and compared to the normal SD rats. Following an oral dose of 600 mg/kg, Cmax and AUC were much lower in RYGB rats compared to normal rats (Cmax: 7.54 *versus* 5.37 μg/mL; AUC: 99.97 *versus* 72.77 μg h/mL for normal and RYGB rats, respectively) indicating a lower absorption of silymarin by RYGB surgery (Chen et al., 2015).

Further studying the effects of these physiological changes post-bypass on oral drug absorption is needed to fully grasp how such changes will affect pharmacokinetics and pharmacodynamics. In the above study, Chen and co-workers developed a novel self-nanoemulsifying drug delivery system (SNEDDS) to enhance the oral delivery of silymarin. The results showed that SNEDDS significantly increased the bioavailability of silibinin, especially in RYGB rats (relative bioavailability of ∼250%). In addition, the novel formulation also increased the Cmax and AUC in RYGB rats compared to the normal rats. The potential reason is that the RYGB procedure delays the interaction between lipolytic enzymes and lipids, which might delay the breakdown of SNEDDS in the intestine (Chen et al., 2015).

### The impact of bariatric surgery on distribution

The volume of distribution (Vd) is usually corrected by the body weight and surface area. Alterations in drug Vd due to both excess weight gain and weight loss may occur, especially for lipophilic drugs, as their Vd depends largely on lipid solubility. In general, the change in Vd does not happen immediately after bariatric surgery, but a substantial decrease in Vd is expected after excess weight loss (Macgregor and Boggs, 1996).

In Rego et al., the article interestingly iterated that the reuptake of technetium-99 m pertechnetate by the liver was significantly lowered in RYGB rats compared to the controlled rats (Rêgo et al., 2010). There are significantly more 99 mTcO4− in the stomach and small intestine in colectomy rats compared to sham and control groups. Thus, this indicates that bariatric surgery might potentially alter tissue distribution.

### The impact of bariatric surgery on metabolism

Bariatric surgery is associated with an improvement in hepatic steatosis and a reduction in glucose production. A vertical sleeve gastrectomy (VSG) was performed on obese mice. Increased insulin clearance was observed in VSG mice, and such an increase was independent of weight loss, suggesting that VSG surgery could change insulin clearance (Ben-Haroush Schyr et al., 2021).

An increase in protein being digested and metabolized was observed due to the alteration in the anatomy of the intestines after bariatric surgery. Dietary nitrogen was recovered in the biliopancreatic limb (BPL) even though the stomach does not expect to transit to the BPL. A hypothesis for this phenomenon is that the 15 N amino acids come from the mesenteric arteria. Thus, remodeling the mucosa in the stomach lining enhances the metabolic pathway (Tessier et al., 2019).

### The impact of bariatric surgery on renal excretion

The excretion of calcium and sodium can also be affected by bariatric surgery. Abegg and co-workers found that renal calcium excretion was increased in RYGB rats (Abegg et al., 2013). Typically, a decrease in intestinal calcium bioavailability would lead to 1,25(OH)2D-mediated upregulation of renal CALB1 and TRPV5 (Transient Receptor Potential Vanilloid 5) expression to prevent and minimize urinary excretion of calcium. However, despite the higher levels of 1,25(OH)2D, urinary excretion of calcium and absolute calcium excretion increased in RYGB rats even though there was no change in renal CALB1 and TRPV5 expression. A possible mechanism is chronic metabolic acidosis in RYGB rats with an increased lactate level (Abegg et al., 2013). Another study found more rapid excretion and less retention of dietary sodium load in rats post-RYGB (Bueter et al., 2012) ^6^. Increases in sodium and water excretion can affect drug serum concentrations and efficacy.

### Effect of bariatric surgery on pharmacodynamics

Pharmacodynamics is the branch of pharmacology that focuses on the relationship between drug concentrations at the site of action and its effects on the body. These effects can range from therapeutic to adverse; both can be dose-dependent or independent. Bariatric surgery creates significant alterations to the physical structure and physiology of the gastrointestinal tract, which undoubtedly alters the pharmacokinetic parameters which have been studied. However, there need to be more studies analyzing the effects of bariatric surgery on pharmacodynamic parameters using rodent models. More studies are needed to fully see bypass procedures’ effect on drug potency and efficacy.

### Clinical relevance of animal models

Animal studies can guide further research that can be translated to human experiments, since the impact of bariatric surgery on the physiology of animals and humans can be similar, such as weight loss, fat mass reduction, metabolic pathways, organ functions, and appetite. However, the data from animal studies cannot always be applied directly to humans qualitatively or quantitatively, so the relevance between animal models to human subjects is essential for the validity of the animal models.

Studies have shown the relevance of physiological mechanisms is more important than the quantitative correlations of the observations. Several studies showed that RYGB and VSG could change animal meal patterns and energy expenditure, leading to significant weight loss (Zheng et al., 2009; Bueter et al., 2012; Laurenius et al., 2012). These mechanisms can be similar in animals and humans since weight loss after surgery is primarily caused by reduced energy intake and meal pattern changes. Negative consequences of RYGB, such as demineralization of the skeletal system, an increased risk of excessive alcohol intake, and fluctuating blood glucose concentrations, were observed in animals. These findings were found in human subjects as well (Hajnal et al., 2012; Thanos et al., 2012). The impact of bariatric surgery on specific metabolic pathways and signaling pathways was similar in both human and animal studies. Bariatric surgery could elevate GLP-1 levels, which could be attributable to post-surgery metabolic effects in rats and humans (Chandarana et al., 2011; Mokadem et al., 2014; Ye et al., 2014). The increase in GLP-1 level could increase insulin release, which may affect the level of counterregulatory hormones. This pathway was also considered to be comparable to crossing species.

The extrapolation of animal data to humans is always challenging since human physiology, anatomy, and lifestyle can differ dramatically. Therefore, clinical trials are necessary to verify the results obtained from animal studies. Nevertheless, animal studies are the more convenient approach to investigate the long-term effects of bariatric surgery on body physiology and evaluate the potential risks and side effects.

## Conclusion and perspectives

With the increasing rates of worldwide obesity, there will undoubtedly be an increase in patients undergoing bariatric surgery for weight reduction and other disease treatments. Bariatric surgery is currently one of the most used procedures in the United States in obese patients with or without obesity-related comorbidities such as type 2 diabetes mellitus (T2DM), hypertension, dyslipidemia, sleep apnea, or other respiratory diseases (Noria and Grantcharov, 2013; Algahtani et al., 2016). Bypass procedures make significant alterations to the gastrointestinal tract that are both physical and physiological, which can, in turn, affect the pharmacokinetic properties of medications. Furthermore, recent studies indicate that surgery significantly alters enterohepatic bile acid circulation and gut microbiota, which may also impact the drug intestinal absorption and metabolism (Yoshino et al., 2020; Tu et al., 2022). This is particularly important as patients undergoing these procedures tend to have several chronic obesity-related conditions that require them to be on medications.

Pharmacokinetic and pharmacodynamic studies using rat model of bariatric surgery can highlight proposed mechanisms of potential alterations in absorption, distribution, metabolism, and excretion, as well as the myriad of physiological changes that can further affect oral drug bioavailability. However, such studies are limited, and few studies investigate the effects of surgeries, such as RYGB, on the pharmacokinetic parameters of medications. To adequately and safely treat patients undergoing such procedures, more studies must be done to investigate these parameters, especially in medications commonly used by this patient population. From the *in vitro* studies provided, the evidence further indicates the significance of the pharmacokinetics alteration that can be used to warrant the investigation within human trials and the correction between bariatric surgery and drug monitoring.
